# Climate change impacts on marine ecosystems through the lens of the size spectrum

**DOI:** 10.1042/ETLS20190042

**Published:** 2019-05-03

**Authors:** Ryan F. Heneghan, Ian A. Hatton, Eric D. Galbraith

**Affiliations:** 1 Institut de Ciència i Tecnologia Ambientals (ICTA-UAB), Universitat Autònoma de Barcelona, 08193 Barcelona, Spain; 2 ICREA, Pg. Lluís Companys 23, 08010 Barcelona, Spain

**Keywords:** climate change, ecosystem measures, marine ecosystem, metabolism, primary production, size spectrum

## Abstract

Climate change is a complex global issue that is driving countless shifts in the structure and function of marine ecosystems. To better understand these shifts, many processes need to be considered, yet they are often approached from incompatible perspectives. This article reviews one relatively simple, integrated perspective: the abundance-size spectrum. We introduce the topic with a brief review of some of the ways climate change is expected to impact the marine ecosystem according to complex numerical models while acknowledging the limits to understanding posed by complex models. We then review how the size spectrum offers a simple conceptual alternative, given its regular power law size-frequency distribution when viewed on sufficiently broad scales. We further explore how anticipated physical aspects of climate change might manifest themselves through changes in the elevation, slope and regularity of the size spectrum, exposing mechanistic questions about integrated ecosystem structure, as well as how organism physiology and ecological interactions respond to multiple climatic stressors. Despite its application by ecosystem modellers and fisheries scientists, the size spectrum perspective is not widely used as a tool for monitoring ecosystem adaptation to climate change, providing a major opportunity for further research.

Given the millions of species living in the global ocean, their diverse life strategies and inter-relationships, and the multiple dimensions of anthropogenic stressors, it can be extremely challenging to grasp the overall impact of climate change on marine ecosystems [1]. In spite of this great complexity, observations have shown that ecosystem size structures tend to be highly regular, with many small and few large individuals, decreasing in abundance with size according to a simple power law distribution. This simple power law relationship is known as the abundance-size spectrum. The size spectrum encompasses all species and has long been known to be among the most robust large-scale regularities in aquatic ecology [2]. As such, it provides a unique lens through which to integrate biotic changes from multiple aspects of climatic change.

Below, we review the physical, biogeochemical and ecological impacts projected by complex numerical models for the remainder of this century, as an illustration of current expectations. We then turn to the size spectrum as a more intuitive, readily grasped framework that provides a bird's eye view of the ecosystem and helps to simplify the expectations, as well as revealing shortfalls in mechanistic understanding.

## Numerical model predictions of impacts on the marine system

**Figure 1.**
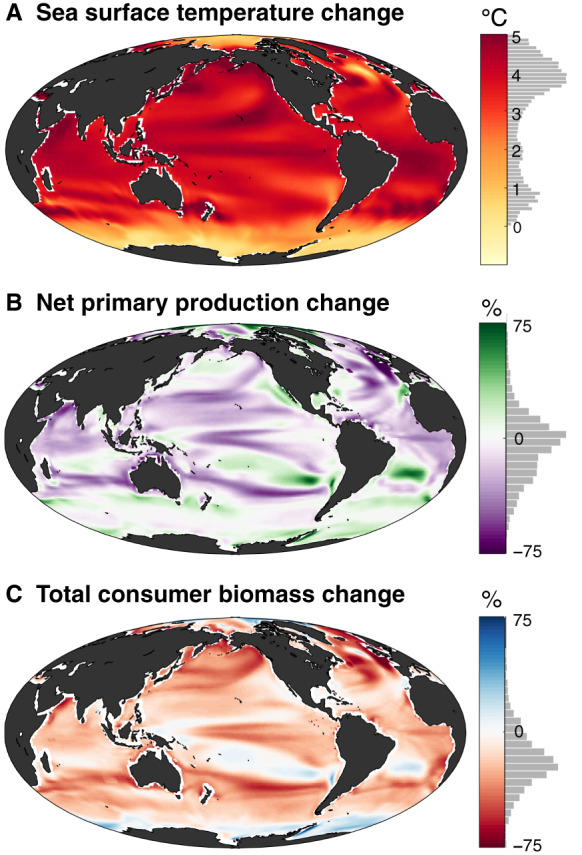
Simulated changes to global marine ecosystems. Model projections of changes in sea surface temperature, production and biomass from 2000 to 2100 under strong emissions (RCP8.5). Grey bars show the frequency distribution of values over the entire ocean. (**A**) The Institut Pierre Simon Laplace (IPSL) climate model projection of sea surface temperature changes. (**B**) The climate model (from **A**) is coupled with a biogeochemical model to project the percentage change in net primary production (NPP). (**C**) Model output from **A** and **B** are used as inputs for consumer size-spectrum models to project the percentage change in total consumer biomass.

Global climate models are increasingly applied as a basis for projecting the impacts of climate change [3–5]. Climate models are computationally intensive, three-dimensional representations of the atmosphere and ocean within which physical equations are solved numerically. These models are used to project climate evolution in response to greenhouse gas emission scenarios. As an example using the Institut Pierre Simon Laplace (IPSL) climate model [4,6], Figure 1A shows the projected change in sea surface temperature over the 21st century under strong emissions (RCP8.5). The model predicts that sea surface temperature will increase virtually everywhere in the ocean as a direct consequence of atmospheric warming, with the strongest warming where shifting ocean currents increase the relative proportion of warmer waters and the least warming at high latitudes where exchange with cold deep waters and/or persistent sea ice keeps the water close to freezing [4].

The IPSL model also includes simple representations of photosynthetic and heterotrophic plankton, which simulate the biogeochemical response to the projected physical climate changes. Figure 1B shows the corresponding response of net primary production (NPP) to the RCP8.5 scenario, showing a mosaic of local increases and decreases of up to 75%, adding up to a global decline of 5%. Climate-biogeochemical models are also used to project other ecosystem stressors, including deoxygenation and acidification of ocean waters [4,7].

Recently, dynamic numerical models of marine animals have been developed that can operate within the same spatial and temporal framework as the climate-biogeochemical models. Figure 1C shows the average change in total consumer biomass of four models from the Fisheries and Marine Ecosystem Model Intercomparison Project (FishMIP) [8,9] when subjected to the same physical and biogeochemical forcings shown in Figure 1A,B. Under this scenario, total consumer biomass is projected to change locally by up to 75% with a spatial pattern determined by the pattern of NPP changes [10], adding up to a global decrease of 20%.

These biogeochemical and animal models provide a current best-guess of the macro-ecological effects of climate change. Yet, they remain relatively rudimentary, in that they typically use a small set of equations to describe physiological rates and trophic relationships across the entire community, neglecting important processes and variation. Increasing model complexity may allow simulation of more aspects of the marine ecosystem but will also make their predictions more difficult to interpret [11,12]. Even a few modelled processes quickly multiply into a large number of parameters, assumptions and interactions, the effect of which is challenging to understand. The tension between additional biological realism and better understanding of the underlying mechanisms requires a hierarchy of perspectives, from complex models to simple representations of ecosystem structure [12,13]. One such simple representation, which can be used to explore the effects of climate change on the structure of the entire marine ecosystem, is the size spectrum.

## The size spectrum

For almost 100 years, the body size of individuals has been recognized as a ‘master’ trait, given its strong link to physiological characteristics across species, and to the role of individuals within ecosystems [14–16]. In marine systems, the vast majority of primary production is carried out by microscopic unicellular phytoplankton, so that the generation of new organic matter from solar energy is undertaken by the smallest individuals. This photosynthetically-derived chemical energy is propagated to animals by predation, which is largely dependent on size relationships, due to gape size limitations and other constraints on the size of prey that a predator can efficiently consume [17,18]. In general, the transfer of energy by predation flows from small to large organisms so that in most aquatic systems, size is a good proxy for trophic level (an organism's position in the food chain). In addition to feeding relations, a great number of individual-level processes have been empirically related to body size through simple scaling relations, from rates of respiration, metabolism, growth, reproduction, and mortality [19,20] to swimming speed, vision and encounter rates [21–23]. The generality of these size-based traits often means that marine organisms at a given developmental stage have much more in common with individuals of the same size regardless of their species than with conspecifics at a different developmental stage [18]. Thus, one can know a lot about a marine organism from its size.

**Figure 2.**
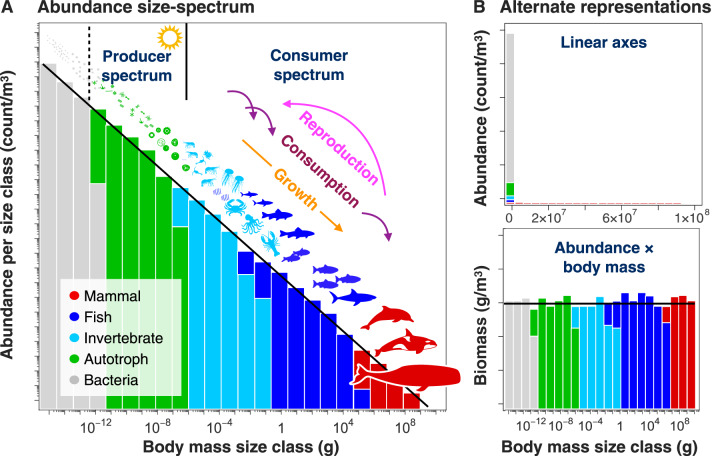
Basic elements of size spectra. (**A**) An idealized size spectrum is shown on log_10_ axes, extending from bacteria to whales. Logarithmically spaced size classes are specified, within each of which the total number of all individuals is summed. The regular linear slope characterizes a power law frequency distribution, shown here with the hypothetical ‘Sheldon’ exponent of −1, implying that abundance is inversely related to body mass. (**B**) Two alternate representations of the same size spectrum. On top, the size spectrum is plotted on linear, rather than logarithmic axes. This representation shows that bacteria are exceedingly abundant, while most size classes include only large mammals, but otherwise, little information is conveyed. A more useful representation is the biomass spectrum (bottom), which is the product of the X and Y axes in **A**, giving the total biomass in logarithmic size classes. An abundance slope of −1 (in **A**) corresponds to a biomass slope of 0.

The size spectrum describes the size-frequency distribution of organisms in an ecosystem. It is constructed by taking the sum of all individuals (regardless of species identity) within logarithmically spaced bins (e.g. total count from 0.1–1 g, from 1–10 g, from 10–100 g, etc.) [2,24,25]. This typically yields a histogram that is strongly right-skewed: very many small individuals, and a few very large individuals (Figure 2B, top). On log–log axes, the relation reveals a straight line extending over many orders of magnitude, called a power law. The size spectrum can thus typically be described by a function of body size *m*, *f*(*m*) ∝ *m^a^*, where the exponent *a* is the slope of the spectrum. As illustrated schematically in Figure 2A, the size spectrum implicitly reflects the outcome of all ecological processes including predation, the growth of individuals through different size classes, and the repopulation of smaller size classes through reproduction [2,24,25]. The size spectrum has many alternative representations, the most common of which is given by multiplying the abundance or frequency of a size class *N* by the mean of the size class *m* to obtain the total biomass *B* in each log bin (*B *= *Nm*; Figure 2B, bottom).

Remarkably, plotting size spectra as logarithmically spaced biomass bins has often revealed an even distribution across portions of the marine ecosystem that is hypothesized to hold from bacteria to whales [26]. This distribution, first suggested by Sheldon and coauthors [26] based on plankton measurements in the open ocean, implies that the numerical abundance of organisms decreases inversely with body size with a slope of −1 when plotted on logarithmic axes (Figure 2A), where the number of individuals in a size class *m* is described with the function *f*(*m*) ∝ *m*^−1^. Since the publication of this hypothesis almost 50 years ago, a large body of empirical work in different ecosystems and for different assemblages of species has largely corroborated the roughly inverse relation between abundance and body size [2]. However, it remains unknown how closely the −1 slope is adhered to across the entire span of marine organisms, or how the distributions may differ in varied coastal ecosystems. Basic theory and a growing family of models have been developed to elucidate the mechanisms underpinning the marine size spectrum [2,24,25,27], and how human impacts such as fishing and climate change may affect its structure [28–34]. Despite these significant advances, we still do not fully understand what basic generative processes are responsible for the regularity of the size spectrum. Intriguingly, a great number of phenomena exhibit power law size-frequency (or rank-frequency) distributions with similar slopes, including large cities, individual wealth, and word use [35].

The size spectrum can be used to represent any portion of the marine ecosystem, including both the coastal and open ocean [28,29,36,37]. Because marine organisms are patchily distributed in space and time, the spectrum will generally show greater regularity (and therefore be more useful) on larger spatial scales, longer timescales and over a larger range of body sizes. On these scales, changes in its shape — namely its slope, elevation and regularity — present a powerful means by which to explore the impacts of climate change on the whole-ecosystem structure.

**Table 1:** Possible effects of climate change on the size spectrum

Physical change	Biological effect	Size spectrum prediction
Increasing ambient water temperature [4]	Increased metabolic rates [20,39,40]	Decreased elevation steeper slope, decreased regularity
	Species distribution shifts [41]	Steeper slope, decreased regularity
Decreasing nutrient supply [42–48]	Decreased primary production and smaller median producer size [49–51]	Decreased elevation, decreased regularity (?)
Novel ecosystems [41,52–55]	Lower trophic efficiency [56]	Steeper slope, decreased regularity (?)
Increasing extreme events [57,58]	Decreased primary production [59] (?)	Decreased elevation
	Decreased trophic efficiency [60] (?)	Steeper slope (?), decreased regularity (?)
Deoxygenation [61]	Decreased metabolic rates [62] (?)	Steeper slope (?)
	Increased anoxia [63] (?)	Decreased regularity (?)
Acidification [64]	Decreased aerobic capacity [65] (?)	Steeper slope (?), decreased regularity (?)

A (?) indicates where there is no apparent consensus in the literature regarding the indicated biological effect, or potential impact on the size spectrum.

## What drives changes in size spectrum shape?

**Figure 3.**
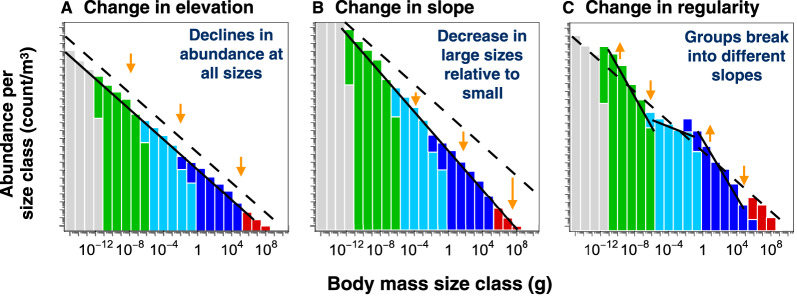
Possible responses of the size spectrum under climate change. The dashed line in each figure is a hypothetical pre-industrial spectrum denoting approximately ‘natural’ conditions. These changes are idealized, to highlight schematic changes to the size spectrum. (**A**) Changes in elevation result from changes that affect all individuals equally (e.g. lower net primary production at the base of food-chain, or uniform temperature-induced metabolism increases across all sizes). (**B**) Changes in slope result from compounding changes that affect coupling across size classes (e.g. a decrease in trophic transfer efficiency would cause larger organisms to decrease more than smaller organisms). (**C**) Changes in regularity result from heterogenous responses to change among different components of the community, or uneven spatial or temporal drivers.

We can classify changes in climatic components according to the kinds of change they elicit in ecological structure (Figure 3). The abundance at the geometric midpoint of the size spectrum, which we refer to as the elevation, will vary with environmental changes that affect all individuals proportionally (e.g. a universal change in mass-specific respiration rate). Changes in slope, on the other hand, are expected from environmental changes that consistently affect the interactions across size classes, so that the effects are compounded with size (e.g. the temperature response of growth or consumption efficiency between size classes; see arrows in Figure 2A) [38]. Finally, changes in regularity are expected from physical changes that differentially affect different taxonomic or functional groups (e.g. different temperature sensitivities of ectotherms and endotherms), or act irregularly in space (geographic distribution shifts) or time (altered phenology or frequency of extreme events). Biotic impacts from climate change are likely to interact, confounding a clear delineation between these different expectations. Nevertheless, the slope, intercept and regularity of the size spectrum provide a means to bridge underlying mechanistic processes with macroecological outcomes. Using these three size spectrum properties, we can use the size spectrum as a simple framework through which to explore the impacts of climate change on the integrated marine ecosystem (Table 1).

### Increasing water temperature

The elevation of the size spectrum would be expected to change (Figure 3A) if the rate at which energy is used across the spectrum varied by a universal temperature-dependent response of metabolism [20,39,40]. Because metabolic rates are generally faster at higher temperatures and marine organisms are predominantly ectothermic (i.e. they do not internally regulate their body temperature), warming would increase the rate of energy consumption through respiration. Faster biomass-specific respiration would thereby decrease the amount of biomass that can be supported for a given amount of energy [66]. Although the general increase in metabolic rates with temperature is not debated, the mechanistic pathways by which temperature affects organism physiology remain unclear, introducing some uncertainty into this prediction [67].

This temperature effect could be mitigated by a shift to organisms with lower inherent metabolic rates [68] or less active lifestyles [69]. It may also be compensated or exacerbated by several other vital rates with similar temperature sensitivity. With these further caveats in mind, we can estimate a drop of 8% in elevation for each 1°C increase in temperature, assuming a slope of −1 and a universal metabolic activation energy (*E_a_*) of 0.6 eV [66]. This should be seen as only one component of the impact of warming, although it is not inconsistent with a recent estimate of a 7 ± 4% decline per 1°C based on a reanalysis of fisheries stock assessments for 235 species from 1930 to 2010 [70], and it does fall within the range projected by FishMIP models [9].

Trophic efficiency, equal to the production rate of a consumer relative to its prey, may change in response to rising temperatures and has been shown experimentally [71,72]. A lower trophic efficiency would reduce the abundance of macroscopic animals in relation to plankton and bacteria [73] since less energy is transferred to larger organisms at each trophic step. The result is trophic amplification [72], which corresponds to a relative decrease in larger organisms and a steeper size spectrum slope (Figure 3B).

Although the Sheldon hypothesis suggests that a relatively regular slope prevailed under pre-industrial conditions, there is no guarantee that it should remain stable during major transitions such as those currently underway (Figure 3C). If a regular slope of the Sheldon spectrum is an optimized outcome to which an ecosystem adapts, then an interval of rapid change could cause breaks in the distribution, as different species and assemblages are impacted in different ways. For instance, mammal endotherms are known to derive an advantage in colder waters [74]. A warmer ocean could potentially diminish this advantage at higher latitudes, contributing to declines in the mammal proportion of the spectrum.

### Decreasing nutrient supply

Changes in NPP are expected to result from alterations of the upward transport of cold, nutrient-rich deep water to large areas of the ocean surface (Figure 1B) [42–45]. Some observations suggest that an expansion of the most nutrient-depleted regions [46,47] and a long-term decline in NPP in the world's oceans [48] have already occurred. Any change of NPP will change the total amount of chemical energy available to support the energetic needs of all organisms within the ecosystem [42]. Since total NPP is roughly proportional to total metabolism (only a very small fraction of NPP escapes respiration to be buried in sediments [75]), this would necessarily affect the total biomass of the entire ecosystem. Thus, all else being equal, the elevation of the size spectrum would fall with a decrease in NPP and vice versa (Figure 3B).

Nutrient-limited conditions also favour smaller phytoplankton [49,50] because they can uptake nutrients faster at low concentrations due to a greater surface area to volume ratio [51]. This could feasibly drive a change in the slope and elevation of the producer spectrum that is decoupled from the response of the consumer spectrum, thus affecting the regularity of the integrated size spectrum (Figure 3C).

### Novel ecosystems

As their current locations become unsuitable, species can maintain their thermal state by migrating to new environments [41], as widely observed [52,53]. However, species move and adapt at different rates [54], altering assemblages and disrupting feeding patterns as preferred prey are no longer available [55]. If such disruption propagates across multiple trophic levels, it could lower trophic efficiency, which would lead to a steeper size spectrum slope and possibly decrease its regularity (Figure 3B,C). For example, forage fish gut content analysis during an exceptional event in the North Pacific (known as the ‘Blob’) showed a switch in zooplankton community composition from lipid-rich taxa to less nutritious, gelatinous zooplankton [56], and a decrease in mean planktivorous fish size and condition, suggestive of decreased trophic efficiency. It has also been argued that climate change drives a change in the timing of the spring bloom relative to egg hatching, resulting in larvae appearing before food is available and impeding the transfer of energy from producers to consumers [76,77].

### Increasing extreme events

Climate change has increased the frequency and intensity of extreme weather events such as heatwaves [57,58]. These events can cause long-term damage, especially to sessile taxa such as coral and kelp, which often have less thermal tolerance than more mobile taxa [78]. Damage to primary producers may lower NPP, lowering the elevation (Figure 3A), while abrupt changes in community composition as a result of heatwaves [59,60], could also increase irregularity in the size spectra (Figure 3C).

### Deoxygenation

Climate change is decreasing the oxygen content of the ocean through the temperature-driven decrease in oxygen solubility, as well as a slowdown of transport from the surface to the deep ocean [61]. There has been relatively little work explicitly linking the impact of deoxygenation on whole-ecosystems, and most studies have looked at individual-level responses. It has been argued that the decline of oxygen together with warming will limit the metabolic scope of animals, by raising metabolic demands at the same time as the oxygen available to support metabolism declines, which may force communities to shift toward organisms with lower metabolic rates [62]. A transition to lower metabolic rates could increase the overall abundance of fish and other ectothermic animals that are able to tolerate the low O_2_. Following similar logic as given above for temperature, this would raise the elevation for this portion of the spectrum. On the other hand, it may also decrease the efficiency of trophic transfer, by limiting motility and feeding, which would steepen the slope (Figure 3B) [79]. Under extreme climate change, large expansions of anoxic waters — in which water-breathing animals cannot survive [63] — would be expected to cause a steeper slope at the upper end of the size spectrum, potentially increasing the irregularity of the ecosystem size spectrum (Figure 3C).

### Acidification

As the ocean absorbs CO_2_ from the atmosphere, it reacts with seawater to form carbonic acid, lowering pH [64]. Ocean acidification is expected to be most severe for calcifying species, which includes important phytoplankton species (e.g. coccolithophores), crustaceans and corals [80]. Studies have also suggested interaction with deoxygenation and temperature, reducing aerobic capacity and reproductive success [65] which could steepen the spectrum slope and/or decrease spectrum regularity (Figure 3B,C). Like deoxygenation, acidification studies have focused mostly on individual, rather than community responses [81], which means there is still a great deal of uncertainty as to how the whole ecosystem will respond to acidification, and how it may change the shape of the size spectrum.

### Non-climate stressors

In addition to climate change, other direct anthropogenic changes will impact marine ecosystem structure. For instance, industrial fishing has caused a tremendous decrease in the abundance of large marine organisms, in some places by more than two orders of magnitude [82,83], truncating the large end of the spectrum. In fact, for many marine ecosystems, fishing pressure is the largest driver of ecosystem structure [84]. Such changes undoubtedly add to, and interact with, impacts from climate change [30,85].

## Concluding remarks and future directions

The size spectrum provides an integrated perspective that could help to better understand how climate change affects the entire marine ecosystem. Due to its simplicity, the size spectrum can complement more complex predictive models by prioritizing processes that dominate ecosystem structure and function and helping to simplify and parameterize these models to accommodate more biological realism. Since abundances vary over small spatial scales and fluctuate on short timescales, the size spectrum is most useful as an integrated measure on large scales, such as for whole ecosystems over many years.

The value of the size spectrum framework for observing changes in ecosystem structure calls for a more systematic collection of abundance-size data across the entire marine ecosystem, from bacteria to whales. This idea is not new; as part of a holistic, ecosystem-level approach to management, fisheries scientists already use size-based indicators such as the spectrum slope as metrics to assess the impact of fishing on ecosystem health [29,36,86,87]. There is already a vast amount of relevant data available, such as >20 years of phytoplankton size spectra collected on the Atlantic Meridional Transect [88]. The continued effort toward systematically unifying existing observations of the marine size spectrum across different scales and taxa could provide a baseline to assess future impacts of climate change and other large-scale human activities on the entire marine ecosystem.

At the same time, the possible geometric changes discussed above highlight the need for better quantification of the underlying mechanisms for how organisms across taxa and size respond to changes in their environment [62,66,89], in order to better tie changes in spectrum shape to changes in climate (Table 1). Even for something as fundamental as temperature, there is not currently a strong fundamental understanding of the most important thermal response mechanisms [67], leaving a large uncertainty in the expected magnitude of change for given warming.

There remain many fundamental questions as to how climate change will affect the marine ecosystem, and a range of perspectives are required to address them. The size spectrum is one available framework that overlooks many details such as changes in species identity and biodiversity, to provide a whole-ecosystem picture. It is our hope that, by illustrating how the complex effects of climate change could be mediated through changes in the slope and elevation of the size spectrum, this review will stimulate wider consideration of the size spectrum framework in ecological climate change research.

## Summary

- The size spectrum provides a valuable perspective on climate change that integrates the large-scale outcome of all interacting mechanisms, providing a contextual backdrop against which to better understand the responses of individual species and changes in biodiversity.
- Changes in the elevation, slope and regularity of the size spectrum provide mechanistic linkages from expected physical changes to macroecological outcomes.
- The value of the size spectrum framework calls for a more systematic collection of abundance-size data across the entire marine ecosystem.
- Developing a better understanding of how the size spectrum will respond to changes in climate requires better quantification of underlying individual physiological responses to multiple stressors, as well as the net results on predation and reproduction.

## Abbreviations

CMIP5: Climate Model Intercomparison Project phase 5
FishMIP: Fisheries and Marine Ecosystem Model Intercomparison Project
IPSL: Institut Pierre Simon Laplace
NPP: net primary production
RCP8.5: Representative Concentration Pathway 8.5
